# Impact of Electronic Health Record Interface Design on Unsafe Prescribing of Ciclosporin, Tacrolimus, and Diltiazem: Cohort Study in English National Health Service Primary Care

**DOI:** 10.2196/17003

**Published:** 2020-10-16

**Authors:** Brian MacKenna, Sebastian Bacon, Alex J Walker, Helen J Curtis, Richard Croker, Ben Goldacre

**Affiliations:** 1 The DataLab Nuffield Department of Primary Care Health Sciences University of Oxford Oxford United Kingdom

**Keywords:** prescribing, primary care, electronic health records, clinical software, branded prescribing, diltiazem, tacrolimus, ciclosporin

## Abstract

**Background:**

In England, national safety guidance recommends that ciclosporin, tacrolimus, and diltiazem are prescribed by brand name due to their narrow therapeutic windows and, in the case of tacrolimus, to reduce the chance of organ transplantation rejection. Various small studies have shown that changes to electronic health record (EHR) system interfaces can affect prescribing choices.

**Objective:**

Our objectives were to assess variation by EHR systems in breach of safety guidance around prescribing of ciclosporin, tacrolimus, and diltiazem, and to conduct user-interface research into the causes of such breaches.

**Methods:**

We carried out a retrospective cohort study using prescribing data in English primary care. Participants were English general practices and their respective EHR systems. The main outcome measures were (1) the variation in ratio of safety breaches to adherent prescribing in all practices and (2) the description of observations of EHR system usage.

**Results:**

A total of 2,575,411 prescriptions were issued in 2018 for ciclosporin, tacrolimus, and diltiazem (over 60 mg); of these, 316,119 prescriptions breached NHS guidance (12.27%). Breaches were most common among users of the EMIS EHR system (breaches in 18.81% of ciclosporin and tacrolimus prescriptions and in 17.99% of diltiazem prescriptions), but breaches were observed in all EHR systems.

**Conclusions:**

Design choices in EHR systems strongly influence safe prescribing of ciclosporin, tacrolimus, and diltiazem, and breaches are prevalent in general practices in England. We recommend that all EHR vendors review their systems to increase safe prescribing of these medicines in line with national guidance. Almost all clinical practice is now mediated through an EHR system; further quantitative research into the effect of EHR system design on clinical practice is long overdue.

## Introduction

Over 1.1 billion prescriptions are issued through primary care in England each year, at a cost of £8.8 billion in 2018 [1]. The overwhelming majority of these prescriptions are generated through an electronic health record (EHR) system [2], where the clinician selects a medicine from a “picking list,” specifies directions (eg, “once daily”), and then signs the prescription either electronically or on a printed copy. All National Health Service (NHS) practices use one of four EHRs made available through NHS Digital [3]. We have previously described a small design choice in an EHR medicines selection screen that costs the NHS approximately £9.5 million in one year across a wide range of drugs [4,5].

Tacrolimus and ciclosporin are used in organ transplantation and other conditions, such as rheumatoid arthritis, psoriasis, and severe atopic dermatitis. Both medicines have a narrow therapeutic window: minor differences in blood levels have the potential to cause graft rejection reactions, and switching between tacrolimus products has been associated with reports of toxicity and graft rejection. Therefore, the Medicines and Healthcare products Regulatory Agency (MHRA) recommends that tacrolimus and ciclosporin are prescribed and dispensed by brand name [6,7]. Similarly, diltiazem—a calcium channel blocker commonly used for conditions such as angina and mild-to-moderate hypertension—should be prescribed and dispensed by brand for preparations containing greater than 60 mg, as different brands of modified-release formulations with over 60 mg diltiazem may not have the same clinical effect [8].

Our team delivers OpenPrescribing.net, a publicly funded and openly accessible data explorer for NHS primary care prescribing, with 135,000 unique users in the past year. OpenPrescribing.net supports bespoke data queries alongside numerous predefined standard measures for safety, cost, and effectiveness, with data shared for every practice in England. Two of these standard measures assess compliance with the guidance to prescribe a branded preparation of ciclosporin, tacrolimus, and diltiazem (over 60 mg). Through our prior knowledge around EHR design, as clinicians and researchers engaged with software development, we suspected there may be a relationship between choice of EHR system and breaches of this safety guidance.

We therefore set out to describe and map variation between practices and their parent clinical commissioning groups (CCGs) in breaches of safe prescribing guidance for ciclosporin, tacrolimus, and diltiazem (over 60 mg), and assess the impact of EHR choice on the proportion of safety breaches. Having identified one EHR system as being strongly associated with safety breaches, we conducted user testing to explore the causes of this association. We aimed to produce a rapid report to support further investigations and any required software modifications to the EHRs, in light of the patient safety risk.

## Methods

### Study Design

This study is a retrospective cohort study in prescribing data from all English NHS general practices and CCGs, complemented with data on EHR deployment from NHS Digital, as well as in user testing of two commonly used EHRs by a senior pharmacist.

### Data Sources

We extracted data from the OpenPrescribing.net database. This imports openly accessible prescribing data from the large monthly files published by the NHS Business Services Authority, which contain data on cost and items prescribed for each month, for every typical general practice and CCG in England since mid-2010 until May 2019 [9]. The monthly prescribing datasets contain one row for each different medication and dose, in each prescribing organization in NHS primary care in England, describing the number of items (ie, prescriptions issued) and the total cost. These data are sourced from community pharmacy claims data and, therefore, contain all items that were dispensed. We extracted all available data for typical general practices, excluding other organizations such as prisons and hospitals, according to the NHS Digital dataset of practice characteristics and excluded practices that had not prescribed at least one item per measure. We excluded all other organizations such as prisons and hospitals. Data on which EHR was deployed in each practice were extracted from a monthly file that is circulated by NHS Digital to interested parties and is available on request [9].

### Prevalence of Safety Breaches by General Practices in Implementation of Brand Prescribing of Ciclosporin, Tacrolimus, and Diltiazem

We measured the number of safety breaches and created practice-level deciles at each month for the measure [10] (see Table 1) of proportion of brand prescribing of ciclosporin, tacrolimus, and diltiazem (over 60 mg).

**Table 1:** OpenPrescribing measures of generic ciclosporin, tacrolimus, and diltiazem prescribing.

Measure	Definition (full technical definitions are available on GitHub [10])
Ciclosporin and tacrolimus	Total items of generic ciclosporin and tacrolimus preparations, as a proportion of total items of all ciclosporin and tacrolimus items. These medicines are grouped due to them having similar uses and covered by similar safety alerts by the Medicines and Healthcare products Regulatory Agency (MHRA).
Diltiazem	Total items of generic diltiazem modified-release preparations, as a proportion of total items of all diltiazem modified-release items (over 60 mg).

### Variation in Safety Breaches by Electronic Health Record

We measured the proportion of safety breaches for prescribing of ciclosporin, tacrolimus, and diltiazem for each of the four principal EHRs.

#### Influence of Principal Electronic Health Record on Breaches of Safety Guidance

We conducted mixed-effects logistic regression to determine the effect size of the EHR used and the extent to which CCG membership and other factors affected these estimates. The independent variable was each prescription as a binary choice between generic and branded, while the main fixed-effect variable was EHR vendor, with CCG membership as a random effect. Other practice factors were selected *a priori* based on their reasonable availability, as well as clinical judgement. These were as follows: percentage of patients over 65 years of age, percentage of patients under 18 years of age, percentage of patients with a long-term health condition, dispensing practice status, single-handed practice status, ruralness or urbanness of practice location, and index of multiple deprivation. Where data for predictor variables were missing, these practices were excluded from the regression.

#### Electronic Health Record System User-Interface Evaluation

One senior pharmacist issued prescriptions in the EMIS and SystmOne computer systems to a test patient and observed the prompts. These two systems hold 95% of the EHR-use market share in general practices in England.

### Software and Reproducibility

Data management was performed using Python, version 3.7 (Python Software Foundation), and Google BigQuery, with analysis carried out using Stata 13.1 (StataCorp) and Python. Data, as well as all code for data management and analysis, is archived online in a public, open access repository on GitHub [11].

### Patient and Public Involvement

Our website, OpenPrescribing.net, is an openly accessible data explorer for all NHS England primary care prescribing data, which receives a large volume of user feedback from professionals, patients, and the public. This feedback is used to refine and prioritize our informatics tools and research activities. Patients were not formally involved in developing this specific study design.

### Ethical Approval

This study uses open, publicly available data and data that are publicly available from NHS Digital on request; therefore, no ethical approval was required.

## Results

### Prevalence of Safety Breaches

In 2018, 316,119 of the 2,575,411 (12.27%) prescriptions for ciclosporin, tacrolimus, and diltiazem (over 60 mg) breached prescribing safety guidance. Of practices that prescribed ciclosporin or tacrolimus at least once, 2241 out of 5439 (41.20%) breached safety guidance, while 5777 practices out of 7186 (80.39%) breached safety guidance with regard to diltiazem. The ratio of safety breaches as a proportion of all prescribing for these items for practices was 12.28% (292,331/2,380,128) (10th-90th percentile range = 0%-47%) for diltiazem and 12.18% (23,788/195,283) (10th-90th percentile range = 0%-67%) for ciclosporin and tacrolimus.

### Variation in Safety Breaches by Electronic Health Record

**Figure 1:**
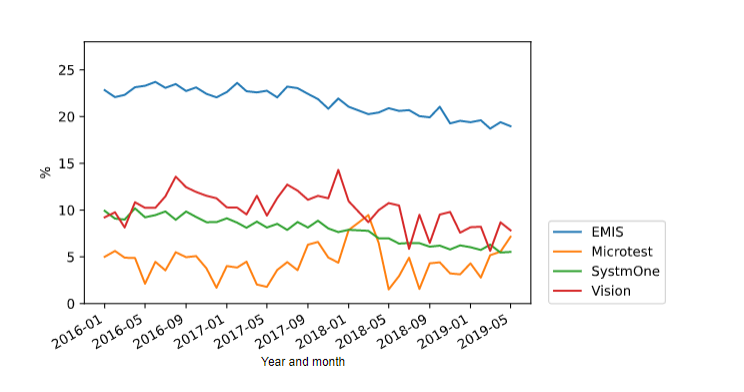
Mean values for the ciclosporin and tacrolimus safety breaches as a proportion of all prescribing by electronic health record (EHR).

**Figure 2:**
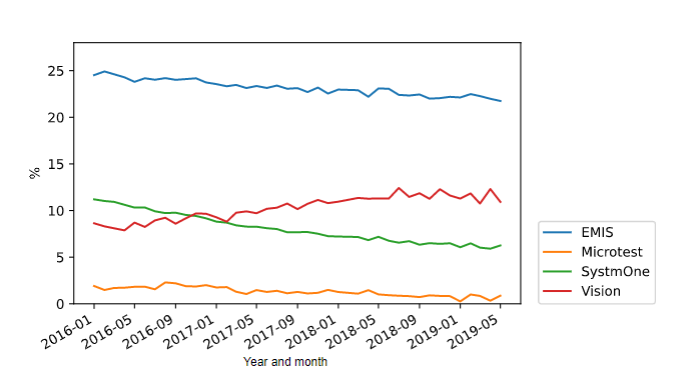
Mean values for the diltiazem safety breaches as a proportion of all prescribing by electronic health record (EHR).

The mean values for the ciclosporin and tacrolimus safety breaches and for the diltiazem safety breaches as a proportion of all prescribing for the respective items from January 2016 to May 2019 are shown in Figures 1 and 2, respectively. The rate of breaches was consistently highest in EMIS practices, and in 2018, 18.81% (16,521/87,828) of all ciclosporin and tacrolimus prescriptions issued from the EMIS EHR system breached safety prescribing safety guidance, while 17.99% (209,371/1,163,578) of all diltiazem (over 60 mg) prescriptions breached safety guidance.

The results of the logistic regression are shown in Table 2. When generating a diltiazem (over 60 mg) prescription, practices using the EMIS EHR system were around 4 times more likely to prescribe in breach of guidance with regard to diltiazem than practices using SystmOne, with a similar size of effect for ciclosporin and tacrolimus breaches. Practices using Microtest and Vision EHR systems were substantially less likely to breach safety guidance, although these practices make up a relatively small proportion of practices. Adjusting for CCG membership as a random effect had little effect on these estimates, although CCG membership was still responsible for a high proportion of the variation in propensity to prescribe generically: 16.4% for diltiazem and 8.4% for ciclosporin. Other factors had even less of an effect on the estimates, though some factors were weakly associated with the propensity to prescribe in breach of guidance (see Multimedia Appendix 1).

**Table 2:** Ciclosporin, tacrolimus, and diltiazem prescribing measures stratified by electronic health record (EHR) system along with odds ratios (ORs) from a univariable and multivariable logistic regression model.

Medicine and EHR systems		Breaches of safety guidance (%), mean	Univariable logistic regression, OR (95% CI)	Mixed-effects logistic regression^a^, OR (95% CI)
**Diltiazem**				
	EMIS	22.7	Reference	Reference
	Microtest	1.0	0.045 (0.039-0.052)	0.051 (0.044-0.060)
	SystmOne	7.0	0.252 (0.249-0.254)	0.239 (0.235-0.243)
	Vision	12.0	0.462 (0.451-0.473)	0.300 (0.290-0.309)
**Ciclosporin and tacrolimus**				
	EMIS	23.2	Reference	Reference
	Microtest	6.1	0.103 (0.072-0.148)	0.161 (0.107-0.242)
	SystmOne	7.8	0.238 (0.230-0.247)	0.271 (0.256-0.287)
	Vision	10.8	0.378 (0.346-0.412)	0.219 (0.195-0.247)

^a^Adjusted for clinical commissioning group (CCG) membership as a random effect, percentage of patients over 65 years of age, percentage of patients under 18 years of age, percentage of patients with a long-term health condition, dispensing practice status, single-handed practice status, ruralness or urbanness of location, and index of multiple deprivation.

### Electronic Health Record System User-Interface Evaluation

#### EMIS

On reviewing the medicines-selection screen, it was observed that when searching for a medicine by its brand name, EMIS always presents the generic version of the medicine as priority in the medicines-picking list; this represents a breach of safety guidance for ciclosporin, tacrolimus, and diltiazem (over 60 mg). Warnings are presented to prescribers to prescribe by brand when a generic version of these medicines is selected; however, these warnings appear alongside multiple other warnings such as interactions with other medications. These warnings take the form of a *pop-up* box, which can be easily overridden allowing the generic version of all three medicines to be issued.

#### SystmOne

When the search terms “ciclosporin,” “tacrolimus,” or “diltiazem” were entered, no results were returned, encouraging a user to search by brand names in line with safety guidance. However, in another part of the user-interface screen there is a separate tick-box, “non-prescribable.” When ticked by a user, input of the search terms “ciclosporin,” “tacrolimus,” or “diltiazem” does return results, allowing selection of the generic medicine in breach of safety guidance. A single, stand-alone *pop-up* is presented if the user proceeds, warning of the importance of brand prescribing. This is in contrast to EMIS, where it is presented alongside other clinical warnings.

## Discussion

### Principal Findings

A total of 2,575,411 prescriptions were issued in 2018 for ciclosporin, tacrolimus, and diltiazem (over 60 mg); of these, 316,119 prescriptions breached NHS safety guidance (12.27%). Breaches were most common among users of the EMIS EHR system (n=3888; December 2018), but breaches were observed in all EHR systems.

### Strengths and Weaknesses

We included all typical practices in England, thus minimizing the potential for obtaining a biased sample. We used prescribing data derived from pharmacy claims data used to calculate the transfer of funds from CCGs to dispensing pharmacies: all parties are motivated to ensure the accuracy of this data. We excluded a small number of settings such as walk-in centers, which typically do not issue repeat prescriptions for medicines, and where no data on EHR usage is available. The data does not distinguish which prescriptions may have been written by hand without EHR generation; however, as 74% of prescriptions are transmitted electronically [2] and many more are generated electronically and then printed on paper, we do not expect this to substantially affect our results. Our data do not include hospital medicines data, but we do not expect the same issue associated with EHR system design to occur in hospitals, as medicines are procured differently and the use of electronic prescribing and EHRs in secondary care in England is limited [12]. Observation of EHR systems was limited to one 30-minute session on each EHR system with limited scenarios in a single location. EHRs can be manually adapted and defaults set locally; compliance with safety guidance may, therefore, differ between individual practices even with the same EHR systems. Additionally, practices may have secondary decision support software systems providing information and prompts, which may affect breaches.

One caveat regarding clinical impact is that the available data reflect prescribing and not dispensing: in England, when medicines are prescribed generically, pharmacies are entitled to dispense a brand, and some pharmacists may try to ensure they dispense the same brand that the patient previously received; however, pharmacists are likely to be blocked in this by lack of access to the patient record and, commonly, by the absence of information on specific brands in the patient’s record [13]. Additionally in England, CCGs employ teams of pharmacy professionals who in some areas may have made concerted manual effort to amend repeat prescriptions following audit. In any case, occasional remedial interventions do not affect the key observation that EHR systems strongly predict safety breaches among prescribing clinicians.

### Findings in Context

We are aware of no prior work on the prevalence of safety breaches around use of ciclosporin, tacrolimus, and diltiazem; however, incomplete implementation of this important national prescribing safety recommendation is consistent with extensive prior work showing incomplete or slow adoption for other national prescribing guidance [14-16]. To our knowledge, we are the first group to use natural variation in prescribing behavior between EHR systems to identify, explain, and address suboptimal prescribing using national data. One small study involving 90 general practices in the Netherlands did find an association between prescribing safety and EHR systems with regard to prescribing of gastroprotective agents to prevent side effects of anti-inflammatory medicines [17]. A 2017 systematic review [18] identified 34 relevant studies exploring the role of computerized systems in suboptimal prescribing. However, none used quantitative methods to compare different systems, instead relying on questionnaires to interrogate clinicians about their experiences of EHRs, qualitative research observing or interviewing clinicians, and descriptions of clinicians’ spontaneous reports on errors and safety issues in EHR systems.

Various small studies have aimed to evaluate the impact of a single specific new change to a computerized prescribing system, as a behavioral intervention to increase the probability of a desired choice being made by clinicians using the system [19-23]. Despite evidence for effectiveness from *pop-ups*, *alert fatigue* has also been described, whereby large numbers of pop-ups can result in salient messages being ignored or disabled by prescribers [24]. One small mixed methods study involving the EMIS EHR system found that only three pop-ups from 117 alerts resulted in the general practitioner checking, but not altering, the prescription [25].

### Policy Implications and Interpretation

Breaches of safety guidance around diltiazem, ciclosporin, and tacrolimus can expose patients to avoidable clinical risk. This is especially important in the case of organ transplantation, where failure to adhere to the safety guidance assessed in our analysis has resulted in organ rejection [7]. The current World Health Organization challenge on medication safety encourages countries to reduce medication errors by 50% by 2022; our finding represents a clear example of an error amenable to change [26]. We strongly recommend that picking-lists be configured in line with best practice guidelines, and that compliance with this be audited by national organizations such as NHS Digital and NHSX. The US National Coordinator for Health Information Technology has made similar recommendations [18,27,28]. For the specific safety issue raised by our analysis, the mandated medicines data standard for the NHS, the Dictionary of Medicines and Devices (dm+d), already includes a field that can be used to mandate branded prescribing where this is required by safety guidance; indeed, it is likely that this field triggers the pop-up warnings described above.

### Future Research

We are concerned by the relative absence of applied practical research around the EHR systems used by clinicians to store information, retrieve relevant information rapidly when assessing a patient, and implement specific clinical actions, such as ordering a test or prescribing a treatment. Health care activity is increasingly computerized, and EHR software is likely to exert a very substantial influence on the way that modern medicine is practiced, in the same way that the rapid explosion in social media usage has changed the ways that people interact socially [29]. We can find no previous attempts to evaluate the impact of EHR system design on clinical practice by analyzing variations between organizations using different EHR systems. In our view, questions of how best to represent, retrieve, and present knowledge about patients to clinicians—and the impact this can have on patient care—should be a key priority for funders and researchers in “digital health.” The NHS and health care systems around the world spend large sums of money on EHR systems [30] and there is substantial room for collaborative improvement to make clinical care safer, cheaper, and more effective. Since sharing our preprint version of this paper, EMIS have implemented a change to their EHR system to address our findings and we will evaluate the effectiveness of this change in 12 months’ time.

There is also a role for open standards in this work. We are aware that in most general practices, complementary decision support systems are deployed alongside the core EHR system, to support medicines-optimization activity and other aspects of quality improvement [31-33]. However, we have been repeatedly blocked from researching the impact of these pop-ups on routine prescribing, as there is no national framework or data detailing which pop-ups are implemented in each setting, and we have been unable to access the data by private negotiation. NHS commissioners and leaders are also blocked from routinely monitoring which pop-ups are implemented across the NHS and from defining or deploying pop-ups nationally. In our view, this represents a failure to set standards and ensure the proportionate and secure data sharing necessary to evaluate and improve patient safety.

### Summary

National guidance on safe prescribing for ciclosporin, tacrolimus, and diltiazem is commonly breached, and the prevalence of safety breaches is strongly influenced by the brand of EHR system. We recommend that EHR vendors immediately review their systems to mandate safe prescribing of these medicines in line with national guidance. We also recommend that more data and funding be made available to support research into the impact of EHR system design on clinical practice.

## Appendix

Multimedia Appendix 1 Full regression models.

## Abbreviations

CCG: clinical commissioning group
dm+d: Dictionary of Medicines and Devices
EHR: electronic health record
ICMJE: International Committee of Medical Journal Editors
MHRA: Medicines and Healthcare products Regulatory Agency
NHS: National Health Service
NIHR: National Institute for Health Research
SPCR: School of Primary Care Research
