# Supplementary figures and images for: Impact of Electronic Health Record Interface Design on Unsafe Prescribing of Ciclosporin, Tacrolimus, and Diltiazem: Cohort Study in English National Health Service Primary Care
Source: J Med Internet Res. 2020 Oct 16;22(10):e17003. doi: 10.2196/17003 (PMC7600019; doi:10.2196/17003)

**Supplementary material: Full regression models**

**Diltiazem**

**
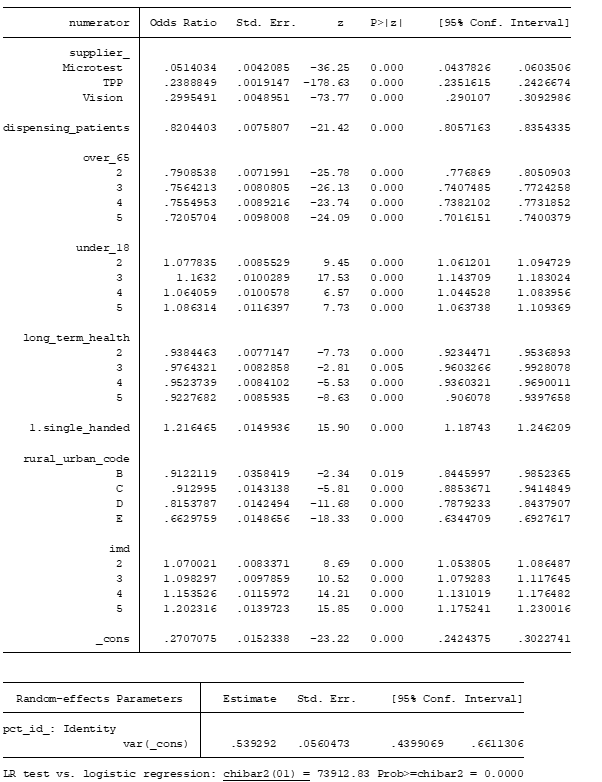
**

**Ciclosporin/tacrolimus**

**
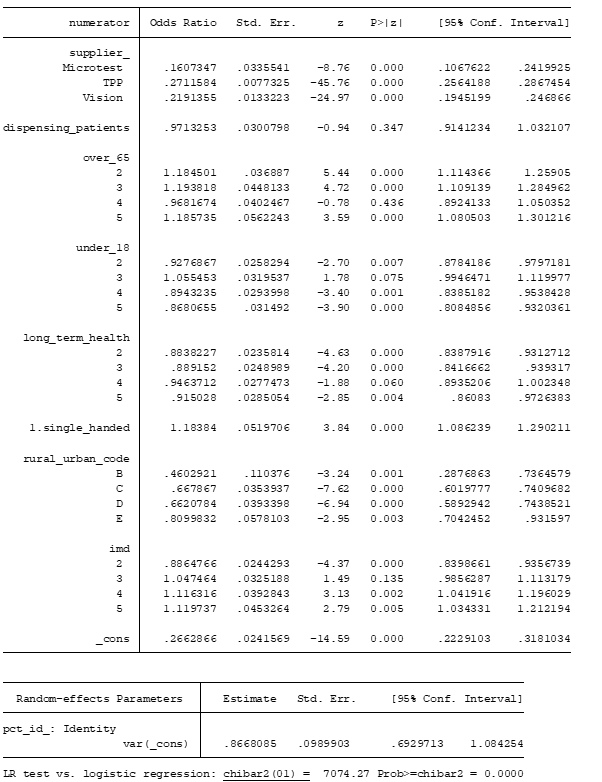
**

Supplement: Multimedia Appendix 1 [file jmir_v22i10e17003_app1.docx]
